# Supplementary material for: The impact of event type and geographical proximity on threat appraisal and emotional reactions to Wikipedia articles
Source: PLoS One. 2020 Jun 1;15(6):e0233770. doi: 10.1371/journal.pone.0233770 (PMC7263619; doi:10.1371/journal.pone.0233770)
Supplement: S1 Appendix — (DOCX) [file pone.0233770.s001.docx]

**Appendix**

This appendix contains the titles of the 30 Wikipedia articles on earthquakes and the 30 Wikipedia articles on terrorist attacks that we used in Study 1.

**30 Wikipedia articles on earthquakes**

2010 Baja California earthquake; 2010 Chile earthquake; 2011 Christchurch earthquake; 2010 Canterbury earthquake; 2009 Fiordland earthquake; 2016 Ecuador earthquake; 2010 Haiti earthquake; October 2015 Hindu Kush earthquake; 2015 Illapel earthquake; 2012 Northern Italy earthquakes; 2016 Taiwan earthquake; 2016 Kumamoto earthquakes; 2009 L'Aquila earthquake; 2011 Lorca earthquake; 2011 Myanmar earthquake; April 2015 Nepal earthquake; 2011 Dalbandin earthquake; 2012 Pernik earthquake; 2010 Pichilemu earthquake; 2009 Samoa earthquake and tsunami; 2010 Serbia earthquake; 2013 Lushan earthquake; 2011 Sikkim earthquake; 2009 Sumatra earthquakes; 2012 Indian Ocean earthquakes; 2012 East Azerbaijan earthquakes; 2011 Tōhoku earthquake and tsunami; 2011 Van earthquake; 2011 Virginia earthquake; 2010 Yushu earthquake.

**30 Wikipedia articles on terrorist attacks**

2015 Ankara bombings; March 2016 Ankara bombing; February 2016 Ankara bombing; 2015 Beirut bombings; Jewish Museum of Belgium shooting; Boston Marathon bombing; 2016 Brussels bombings; Bacha Khan University attack; Garissa University College attack; 2016 Grand-Bassam shootings; June 2016 Istanbul bombing; January 2016 Istanbul bombing; March 2016 Istanbul bombing; 2016 Jakarta attacks; April 2016 Kabul attack; Metrojet Flight 9268; 2015 Kuwait mosque bombing; 2016 Lahore suicide bombing; 2011 Norway attacks; Charlie Hebdo shooting; November 2015 Paris attacks; 2014 Peshawar school massacre; 2016 Pathankot attack; 2015 Sousse attacks; 2015 Sarajevo shooting; 2015 Suruç bombing; 2015 Thalys train attack; Bardo National Museum attack; Westgate shopping mall attack; Zvornik police station shooting.
