# Supplementary material for: The impact of event type and geographical proximity on threat appraisal and emotional reactions to Wikipedia articles
Source: PLoS One. 2020 Jun 1;15(6):e0233770. doi: 10.1371/journal.pone.0233770 (PMC7263619; doi:10.1371/journal.pone.0233770)

PLOS ONE

The impact of event type and geographical proximity on threat appraisal and emotions in Wikipedia articles

--Manuscript Draft--

**Manuscript Number:**	PONE-D-19-26386
**Article Type:**	Research Article
**Full Title:**	The impact of event type and geographical proximity on threat appraisal and emotions in Wikipedia articles
**Short Title:**	Threat appraisal and emotions in Wikipedia articles
**Corresponding Author:**	Hannah Greving<br>Leibniz-Institut fur Wissensmedien / Knowledge Media Research Center<br>Tübingen, GERMANY
**Keywords:**	Emotions; Wikipedia; threat appraisal; anger; geographical proximity
**Abstract:**	The online encyclopedia Wikipedia strives for the objectivity of its content. Simultaneously, Wikipedia provides articles about negative events (e.g., earthquakes, terrorist attacks) which likely elicit strong, negative emotions. These emotions might slip into Wikipedia articles. Previous research has demonstrated that Wikipedia articles on terrorist attacks contained more anger-related content than Wikipedia articles on earthquakes. This previous research used an automatic linguistic analysis tool that counts the number of emotion-related words. In order to extend this research, the first aim of the present research was to replicate these findings by using a different, coding approach. Second, previous research did not look at the geographical location of the negative events, which may be a relevant, influential factor. Emotional reactions may be stronger for geographically closer events (i.e., Europe) than for geographically more distant events (i.e., Asia). Two studies, one with few coders coding many Wikipedia articles (Study 1) and another with many coders coding few Wikipedia articles (Study 2), demonstrated that Wikipedia articles on terrorist attacks elicited more threat, anger, sadness, and anxiety than Wikipedia articles on earthquakes. These effects occurred for negative events in Europe but were absent for events in Asia, with one exception. The anger effect was the same across Europe and Asia. Thus, event type and geographical proximity are relevant factors for explaining threat and emotions in Wikipedia.
**Order of Authors:**	Hannah Greving
	Joachim Kimmerle
**Additional Information:**	
**Question**	**Response**
**Financial Disclosure**<br><br>Enter a financial disclosure statement that describes the sources of funding for the work included in this submission. Review the submission guidelines for detailed requirements. View published research articles from *PLOS ONE* for specific examples.<br><br>This statement is required for submission and **will appear in the published article** if the submission is accepted. Please make sure it is accurate.	The authors received no specific funding for this work.

Powered by Editorial Manager® and ProduXion Manager® from Aries Systems Corporation

**Unfunded studies**<br>Enter: *The author(s) received no specific funding for this work.*<br><br>**Funded studies**<br>Enter a statement with the following details:<br>• Initials of the authors who received each award<br>• Grant numbers awarded to each author<br>• The full name of each funder<br>• URL of each funder website<br>• Did the sponsors or funders play any role in the study design, data collection and analysis, decision to publish, or preparation of the manuscript?<br>• **NO** - Include this sentence at the end of your statement: *The funders had no role in study design, data collection and analysis, decision to publish, or preparation of the manuscript.*<br>• **YES** - Specify the role(s) played.<br><br>* typeset	
**Competing Interests**<br><br>Use the instructions below to enter a competing interest statement for this submission. On behalf of all authors, disclose any competing interests that could be perceived to bias this work—acknowledging all financial support and any other relevant financial or non-financial competing interests.<br><br>This statement **will appear in the published article** if the submission is accepted. Please make sure it is accurate. View published research articles from *PLOS ONE* for specific examples.	The authors have declared that no competing interests exist.

Powered by Editorial Manager® and ProduXion Manager® from Aries Systems Corporation

**NO authors have competing interests**<br><br>Enter: *The authors have declared that no competing interests exist.*<br><br>**Authors with competing interests**<br><br>Enter competing interest details beginning with this statement:<br><br>*I have read the journal's policy and the authors of this manuscript have the following competing interests: [insert competing interests here]*<br><br>* typeset	
**Ethics Statement**<br><br>Enter an ethics statement for this submission. This statement is required if the study involved:<br><br>• Human participants<br>• Human specimens or tissue<br>• Vertebrate animals or cephalopods<br>• Vertebrate embryos or tissues<br>• Field research<br><br>Write "N/A" if the submission does not require an ethics statement.<br><br>General guidance is provided below. Consult the submission guidelines for detailed instructions. **Make sure that all information entered here is included in the Methods section of the manuscript.**	The studies were reviewed by the local ethics committe of the Leibniz-Institut für Wissensmedien. The approval number is LEK 2016/046. We received written consent from participants and used a standard consent form to inform participants about the study. Yet, participants' data was analyzed anonymously.

Powered by Editorial Manager® and ProduXion Manager® from Aries Systems Corporation

**Format for specific study types**<br><br>**Human Subject Research (involving human participants and/or tissue)**<br>• Give the name of the institutional review board or ethics committee that approved the study<br>• Include the approval number and/or a statement indicating approval of this research<br>• Indicate the form of consent obtained (written/oral) or the reason that consent was not obtained (e.g. the data were analyzed anonymously)<br><br>**Animal Research (involving vertebrate animals, embryos or tissues)**<br>• Provide the name of the Institutional Animal Care and Use Committee (IACUC) or other relevant ethics board that reviewed the study protocol, and indicate whether they approved this research or granted a formal waiver of ethical approval<br>• Include an approval number if one was obtained<br>• If the study involved *non-human primates*, add *additional details* about animal welfare and steps taken to ameliorate suffering<br>• If anesthesia, euthanasia, or any kind of animal sacrifice is part of the study, include briefly which substances and/or methods were applied<br><br>**Field Research**<br><br>Include the following details if this study involves the collection of plant, animal, or other materials from a natural setting:<br>• Field permit number<br>• Name of the institution or relevant body that granted permission	
**Data Availability**<br><br>Authors are required to make all data underlying the findings described fully available, without restriction, and from the time of publication. PLOS allows rare exceptions to address legal and ethical concerns. See the PLOS Data Policy and FAQ for detailed information.	Yes - all data are fully available without restriction

A Data Availability Statement describing where the data can be found is required at submission. Your answers to this question constitute the Data Availability Statement and **will be published in the article**, if accepted.<br><br>**Important:** Stating 'data available on request from the author' is not sufficient. If your data are only available upon request, select 'No' for the first question and explain your exceptional situation in the text box.<br><br>Do the authors confirm that all data underlying the findings described in their manuscript are fully available without restriction?	
**Describe where the data may be found in full sentences. If you are copying our sample text, replace any instances of XXX with the appropriate details.**<br><br>• If the data are **held or will be held in a public repository**, include URLs, accession numbers or DOIs. If this information will only be available after acceptance, indicate this by ticking the box below. For example: *All XXX files are available from the XXX database (accession number(s) XXX, XXX.*).<br>• If the data are all contained **within the manuscript and/or Supporting Information files**, enter the following: *All relevant data are within the manuscript and its Supporting Information files.*<br>• If neither of these applies but you are able to provide **details of access elsewhere**, with or without limitations, please do so. For example:<br><br>*Data cannot be shared publicly because of [XXX]. Data are available from the XXX Institutional Data Access / Ethics Committee (contact via XXX) for researchers who meet the criteria for access to confidential data.*<br><br>*The data underlying the results presented in the study are available from (include the name of the third party*	All data is momentarily stored in the data repository at the Leibniz-Institut für Wissensmedien. Upon acceptance, all data files will be available from an open access database.

and contact information or URL).<br>• This text is appropriate if the data are owned by a third party and authors do not have permission to share the data.<br><br>* typeset	
Additional data availability information:	Tick here if the URLs/accession numbers/DOIs will be available only after acceptance of the manuscript for publication so that we can ensure their inclusion before publication.

Powered by Editorial Manager® and ProduXion Manager® from Aries Systems Corporation

Manuscript

Click here to download Manuscript manuscript emotions in wikipedia.docx

The impact of event type and geographical proximity on threat appraisal and emotions in Wikipedia articles

Hannah Greving[1,*] & Joachim Kimmerle[1, 2]

[1] Knowledge Construction Lab, Leibniz-Institut für Wissensmedien, Tübingen, Germany

[2] Department of Psychology, Eberhard Karls University of Tübingen, Tübingen, Germany

* Corresponding author

E-mail: h.greving@iwm-tuebingen.de (HG)

Abstract

The online encyclopedia Wikipedia strives for the objectivity of its content. Simultaneously, Wikipedia provides articles about negative events (e.g., earthquakes, terrorist attacks) which likely elicit strong, negative emotions. These emotions might slip into Wikipedia articles. Previous research has demonstrated that Wikipedia articles on terrorist attacks contained more anger-related content than Wikipedia articles on earthquakes. This previous research used an automatic linguistic analysis tool that counts the number of emotion-related words. In order to extend this research, the first aim of the present research was to replicate these findings by using a different, coding approach. Second, previous research did not look at the geographical location of the negative events, which may be a relevant, influential factor. Emotional reactions may be stronger for geographically closer events (i.e., Europe) than for geographically more distant events (i.e., Asia). Two studies, one with few coders coding many Wikipedia articles (Study 1) and another with many coders coding few Wikipedia articles (Study 2), demonstrated that Wikipedia articles on terrorist attacks elicited more threat, anger, sadness, and anxiety than Wikipedia articles on earthquakes. These effects occurred for negative events in Europe but were absent for events in Asia, with one exception. The anger effect was the same across Europe and Asia. Thus, event type and geographical proximity are relevant factors for explaining threat and emotions in Wikipedia.

Keywords: emotions; Wikipedia; threat appraisal; anger; geographical proximity

Introduction

The online encyclopedia Wikipedia is one of the most popular online services in order to gain access to huge amounts of reliable information (www.alexa.com). Besides factual information, Wikipedia is also a source of information about newly happened events, as anyone can contribute to Wikipedia anywhere and at any time. Devastating, negative events (e.g., earthquakes, terrorist attacks) belong to one category of these newly happened events. Although Wikipedia strictly strives for unbiased, objective, and neutral information on its platform (https://en.wikipedia.org/wiki/Wikipedia:Neutral_point_of_view), such negative events have a high potential for eliciting strong emotions. Therefore, previous research has investigated whether Wikipedia articles differ in their emotional content [1-5]. This research found that Wikipedia articles on man-made attacks (e.g., terrorist attacks) contained more anger-related content than Wikipedia articles on nature-made disasters (e.g., earthquakes).

Yet, these previous studies leave venues for further research. The previous results were mostly based on the mere counts of words of the automatic linguistic text analysis tool Linguistic Inquiry and Word Count (LIWC [6]). It remains unclear whether the effects found with LIWC are also present when assessing complex, felt emotions, and whether the emotional content in the Wikipedia articles also elicits similar emotional reactions in its readers. Moreover, the previous research mostly analyzed English Wikipedia articles and did not account for the country where the negative event had happened. Yet, events that occur in close geographical proximity (e.g., in Europe for a European audience) may elicit stronger emotions than events that occur geographically further away (e.g., in Asia). Therefore, the current research coded the emotional content of existing Wikipedia articles — with either few coders coding many Wikipedia articles or many coders coding few Wikipedia articles — and controlled for the continent where the event had happened in a within-groups experiment. Thus, this research extends previous research in important ways and sheds new light on factors that are relevant for the elicitation of emotions by Wikipedia articles.

Wikipedia and its characteristics

Wikipedia is the most frequently used online platform for acquiring information and, since its founding in 2001, has been growing ever since: It exists in more than 300 languages, of which the English Wikipedia is the most comprehensive one with more than 5.9 million articles (https://meta.wikimedia.org/wiki/List_of_Wikipedias). Thus, Wikipedia provides a huge information and knowledge database which is established by the effective and collective collaboration of masses of individuals on millions of articles [7]. In order to provide high quality information, Wikipedia has strict guidelines to guarantee the provision of unbiased, objective, and neutral information (https://en.wikipedia.org/wiki/Wikipedia:Neutral_point_of_view; [8-10]).

Despite these guidelines, Wikipedia articles may contain emotional content. Such emotional content is likely to be present in Wikipedia for three reasons. First, devastating, negative events such as earthquakes and terrorist attacks are the subject of numerous Wikipedia articles. Especially newcomers to the community of the Wikipedia users often collaborate on articles on newly happened negative events and may not be altogether used to the guidelines of Wikipedia [9, 11-12]. Thereby, emotions may subtly slip into the articles. Second, emotions are core to human beings and influence a wide array of aspects of human functioning and behavior [13]. In particular, they influence information processing [14-15], for example, the production of information [16-17], which is relevant for writing Wikipedia articles. Third, and related to the previous point, it is known that characteristics of Internet users can spill over into Wikipedia and its content [18-20]. Research has shown, for example, that attitudes, opinions, prior knowledge, and group memberships were reflected in written Wiki texts [18, 21-23]. In that sense, emotions as subjective experiences and, thus, characteristics of Internet users are likely to spill over into Wikipedia’s content in the same way.

Emotional content in Wikipedia articles

Previous research has investigated whether Wikipedia articles about different negative events differ in their emotional content [2-4]. This research used appraisal theories of specific emotions [24-26] to explain the elicitation of certain emotions by certain negative events and

used man-made attacks (e.g., terrorist attacks) and natural disasters (e.g., earthquakes) as negative events. In particular, terrorist attacks were expected to elicit more anger, as they occur due to the blameworthy actions of extremists, while earthquakes were expected to elicit more sadness, as they occur due to natural factors and cannot be undone [24-26] (for more elaboration on these expectations see [4]). In line with these theories, the previous research found that Wikipedia articles on terrorist attacks contained more anger-related content than Wikipedia articles on earthquakes, whereas Wikipedia articles on earthquakes contained more sadness-related content than Wikipedia articles on terrorist attacks [2-4].

The results for anger-related content were found both for collectively written, existing Wikipedia articles that were directly assessed from the platform [3-4], as well as for individually written Wikipedia texts that participants produced in controlled laboratory settings [2]. These laboratory experiments also found that man-made attacks, such as shooting rampages and terrorist attacks, elicited more threat appraisal—that is, feelings of being unable to deal with a demanding situation [27]—than nature-made disasters, such as floods and earthquakes. Moreover, threat appraisal was a mediator that could explain the effects on anger and anger-related content in Wikipedia articles. These findings are in line with considerations of terrorist attacks as threatening events [28-30].

In these previous studies, the Wikipedia articles or texts were analyzed with the automatic linguistic text analysis software Linguistic Inquiry and Word Count (LIWC [6]). This software is the most frequently used text analysis tool [31-34]. It is based on the dictionary of a certain language and counts the number of words which belong to certain categories that had been defined by linguistic experts beforehand. One category includes the negative emotion categories anger, sadness, and anxiety, and these were used in the previous research. Although widely used, LIWC can be criticized for being based merely on the counts of words, without being able to assess broader measures of the complex categories it assesses. Thus, the results obtained in previous research for the emotional content in the Wikipedia articles may not completely hold, because LIWC may not entirely capture anger, sadness, and anxiety as complex emotional reactions. It is also unclear whether individuals reading the Wikipedia articles react

emotionally in the same way as the authors who wrote them. We argue that this would be the case, because the emotional reactions would be elicited by the situation itself [24-26].

Another question that could be drawn from previous research is whether the geographical location (e.g., the continent) of the negative event should be considered. In this previous research, only English Wikipedia articles were analyzed. This does not allow for taking into account how close or distant the negative event had been for the Internet users who contributed to these articles. From other research, it is known that the geographical proximity of a negative event is quite relevant for the reaction to the event and for its coverage in the media. That is, close events elicited strong emotions [35] which may be amplified by more intense reporting about close events than distant events [36-38]. In this sense, investigating the geographical proximity of an event is a relevant factor for the elicitation of emotions, with closer events eliciting stronger emotions than more distant events.

The current research

The aim of this research was to shed new light on the elicitation of emotions and threat appraisal by Wikipedia articles. In particular, this research used a coding approach in two studies, each with a different focus. Study 1 was a classical coding study in which four coders rated 60 existing Wikipedia articles on earthquakes and terrorist attacks. As a conceptual extension, in Study 2, 35 participants serving as independent coders indicated their emotional reactions after reading four existing Wikipedia articles on earthquakes and terrorist attacks. Moreover, Study 2 used an Asian and a European earthquake as well as an Asian and a European terrorist attack in order to take the geographical proximity of the negative event into account. Thus, the studies of the current research complement each other by focusing either on many articles with a few coders or a few articles with many coders, and by shifting the coding focus from coding the text to capturing emotional reactions after reading.

Based on the theoretical considerations outlined above and on the findings of previous research, we stated the following hypotheses regarding main effects of type of negative event:

Wikipedia articles on terrorist attacks elicit more threat appraisal than Wikipedia articles on earthquakes (*Hypothesis 1*). Wikipedia articles on terrorist attacks elicit more anger than Wikipedia articles on earthquakes (*Hypothesis 2*). Wikipedia articles on earthquakes elicit more sadness than Wikipedia articles on terrorist attacks (*Hypothesis 3*). As an open research question, we also examined the impact of the type of negative event on anxiety (for more elaboration on this point see [4]).

Moreover, we stated the following hypotheses regarding interaction effects between type of negative event and geographical proximity:

When they occurred geographically closer by, terrorist attacks and earthquakes differ more strongly in their elicitation of threat appraisal (*Hypothesis 4a*), anger (*Hypothesis 4b*), and sadness (*Hypothesis 4c*) than when they occurred geographically further away. As an open research question, we also considered whether there are respective effects for anxiety (for more elaboration on this point see [4]).

Study 1 investigated 30 existing Wikipedia articles on earthquakes and 30 existing Wikipedia articles on terrorist attacks and tested Hypotheses 1–3. The elicitation of emotions while reading the articles was measured by having four independent coders rate their subjective emotional reactions during reading.

Study 1

Method

Design, power analysis, materials, and procedure

This study had a one-factorial between-groups design with two conditions (type of negative event: earthquakes vs. terrorist attacks). We conducted an a priori power analysis with G*Power [39] for a one-way analysis of variance (ANOVA) with two groups with a medium to large effect size of $\rho = 0.40$, a power of 0.80, and an alpha level of $\alpha = 0.05$. This analysis showed that we would need a total sample size of 52 Wikipedia articles. Following these calculations, we used 30 Wikipedia articles about earthquakes and 30 Wikipedia articles about terrorist attacks

(see Appendix; see also [4]). The selection criteria for these articles were that they dealt with the respective event, were available in English, and did not have occurred earlier than 2001, because Wikipedia has only existed since then. Another criteria relevant for emotional reactions was that the article was initially created immediately after the event had happened. From these 30 articles about earthquakes and 30 articles about terrorist attacks, we only used the main text and excluded the tables, figures, notes, references, and external links in order to prevent for confounds stemming from material different from the main article.

In order to code the emotionality of the articles, we used four independent coders. Their task was to read each article and rate their own emotional reactions while reading. In particular, they were instructed to spontaneously indicate their subjective emotional reactions after each large paragraph within the Wikipedia articles. On average, the articles had $M = 5.63$ paragraphs ($SD = 2.35$, range: 1 – 11). For each coder, the ratings per paragraph were later averaged across each article. Regarding the emotional reactions, the four coders indicated how threatened ($\alpha = .27$), angry ($\alpha = .76$), sad ($\alpha = .55$), and anxious ($\alpha = .42$) they felt on a 9-point Likert scale ranging from 1 (*not at all*) to 9 (*very much*).

Results

We tested whether Wikipedia articles about earthquakes and terrorist attacks differed in the emotional reactions they elicited while the raters were reading them. To do so, we conducted separate one-way ANOVAs with negative event (earthquakes vs. terrorist attacks) as between-groups factor for threat, anger, sadness, and anxiety. The results revealed that there was an effect for *threat*, $F(1, 58) = 12.75$, $p = .001$, $\eta_p^2 = .180$. Wikipedia articles about terrorist attacks elicited more feelings of threat ($M = 4.39$, $SD = 0.70$) than Wikipedia articles about earthquakes ($M = 3.68$, $SD = 0.84$; Fig 1 on the far left), which supported Hypothesis 1. The effect for *anger* was significant as well, $F(1, 58) = 197.45$, $p < .001$, $\eta_p^2 = .773$. Wikipedia articles about terrorist attacks elicited more anger ($M = 3.79$, $SD = 0.69$) than Wikipedia articles about earthquakes ($M = 1.53$, $SD = 0.55$; Fig 1 second from the left), which supported Hypothesis 2. There was also a significant effect for *sadness*, $F(1, 58) = 11.64$, $p = .001$, $\eta_p^2 = .167$. Wikipedia

articles about terrorist attacks elicited more sadness (*M* = 4.12, *SD* = 0.70) than Wikipedia articles about earthquakes (*M* = 3.42, *SD* = 0.87; Fig 1 second from the right), which was opposite to Hypothesis 3. Finally, the effect for *anxiety* was significant as well, F (1, 58) = 18.98, $p < .001$, $\eta_p^2 = .247$. Wikipedia articles about terrorist attacks elicited more anxiety (*M* = 2.95, *SD* = 0.69) than Wikipedia articles about earthquakes (*M* = 2.21, *SD* = 0.61; Fig 1 on the far right).

Fig 1. Means and standard errors for the dependent variables threat, anger, sadness, and anxiety for the Wikipedia articles on earthquakes and the Wikipedia articles on terrorist attacks of Study 1.

Discussion

The findings of Study 1 demonstrated that reading Wikipedia articles on terrorist attacks elicited more feelings of threat, anger, sadness, and anxiety than reading Wikipedia articles on earthquakes. The findings for threat and anger are in line with previous research [2-4]. One could argue that although many Wikipedia articles were coded, the results of Study 1 were based on a relatively small number of raters and partly suboptimal reliabilities. In order to improve our method, we used more elaborate measurements for threat and the three emotions and changed the following aspects in Study 2: (1) We used only two Wikipedia articles on earthquakes and two Wikipedia articles on terrorist attacks, (2) we used more coders, that is, 35 participants who rated these articles and used a within-groups design, (3) we instructed participants to indicate their emotional reactions after reading the whole Wikipedia article, and (4) we used a commonly used appraisal measure for threat and assessed the emotions with three items each.

Moreover, we added geographical proximity (i.e., the location where the negative event had happened) as a factor to our study design. The study was conducted in Germany, which means that negative events happening in Europe were geographically closer than negative events happening in Asia. Consequently, the type of a negative event (i.e., an earthquake or a

terrorist attack) should have a greater impact when the events occurred in Europe compared to when they occurred in Asia. Thus, in Study 2, we tested Hypotheses 1–3 and Hypotheses 4a–4c.

Study 2 used four existing Wikipedia articles about an earthquake in Asia, an earthquake in Europe, a terrorist attack in Asia, and a terrorist attack in Europe. We investigated the emotional reactions of participants after they had read each one of the four articles and used a complete within-groups design.

Study 2

Method

Design, power analysis, and participants

The study had a 2 (type of negative event: earthquake vs. terrorist attack) × 2 (geographical proximity: Asia vs. Europe) within-groups design. We conducted an a priori power analysis with G*Power [39] for a repeated measures ANOVA with four measurement points with a medium to large effect size of $\rho = 0.40$, a power of 0.80, and an alpha level of $\alpha = 0.05$. This analysis showed that we would need a total sample size of 10 participants. We followed these calculations, but simultaneously had to adjust to the conditions in the laboratory and the availability of participants who signed up for the study. Therefore, 35 participants (26 female, 9 male; $M_{age} = 26.91$, $SD = 9.94$) who had German as mother tongue participated in this study. The study lasted approximately 20 minutes and was conducted as the second study of a one-hour lab session. For their participation in the entire one-hour lab session, participants received €8 (approximately $9) as compensation.

Procedure and materials

The study was conducted in a laboratory. This laboratory comprised six private cubicles which were soundproof and each equipped with a computer. The study was conducted with the computers and, thus, all instructions and measures were displayed on the computer screens. After giving informed consent, all participants read four existing German Wikipedia articles on negative events. These articles only contained the main text and did not include figures, tables,

references, external links, or other information unrelated to the main text. The criteria for the selection of the articles were the same as in Study 1, but we applied the following additional criteria: The articles were available in German, the lengths of the main texts were comparable across geographical location, and the event had happened in the last few years. Finally, we used the following articles: April 2015 Nepal earthquake (earthquake in Asia), August 2016 earthquake in Italy (earthquake in Europe), January 2016 Bacha Khan University attack (terrorist attack in Asia), and June 2017 London Bridge attack (terrorist attack in Europe).

Roughly half of the participants (N = 18) first read the two earthquakes articles and then the two terrorist attacks articles, while the other half of the participants (N = 17) first read the two terrorist attacks articles and then the two earthquakes articles. We checked whether this difference in the order of articles had an influence on the dependent variables. To do so, we conducted four mixed ANOVAs with order of articles as between-groups factor and the four ratings of the articles of each dependent variable (i.e., threat appraisal, anger, sadness, and anxiety) as within-groups factor. These analyses did not reveal any significant main effect of order of articles on the dependent variables, all F's $(1, 33) < 1$, *ns*. Therefore, we had excluded the order factor from the analyses.

After reading each Wikipedia article, participants, first, indicated how threatening they appraised the event described in the Wikipedia article and, then, which emotions they felt concerning this event. To make sure that participants would leave the laboratory in a balanced state of mood, they thought about a current positive situation in their lives at the end of the study and wrote some sentences about it. Finally, they were carefully debriefed, received their compensation, and were dismissed.

Measures

Threat appraisal was measured with two items. The first item asked participants “How demanding is the negative event in the article you just read for you?” and assessed their answers on a 9-point Likert scale ranging from 1 (*not demanding at all*) to 9 (*very demanding*). The second item asked participants “How well can you deal with the negative event?” and assessed

their answers on a 9-point Likert scale ranging from 1 (*not at all*) to 9 (*very well*). These items are typical items often used in research about threat [27, 40]. We then calculated a difference score of the two items with higher scores indicating more threat appraisal.

As *emotions*, we measured with three items each *anger* (hostile, angry, annoyed, $\alpha = .77$), *sadness* (disappointed, sad, depressed, $\alpha = .94$), and *anxiety* (scared, afraid, frightened, $\alpha = .94$; [41]) regarding the negative event in the article. All items were assessed on a 9-point Likert scale ranging from 1 (*does not apply at all*) to 9 (*applies very much*) and were preceded by the words "When I am thinking about the negative event in the article, I am/feel …".

Results

In order to test whether the four articles differed in their elicitation of threat appraisal, anger, sadness, and anxiety, we conducted repeated measurement ANOVAs with type of negative event (earthquake vs. terrorist attack) and geographical proximity (Asia vs. Europe) as within-groups factors. We conducted these ANOVAs for each of the dependent variables in the same way and, in the following, present the results of these analyses for each variable.

Threat appraisal

The results revealed a significant main effect of type of negative event, $F\ (1, 34) = 5.64$, $p = .023$, $\eta_p^2 = .142$. The terrorist attacks elicited more threat appraisal ($M = 0.50$, $SD = 2.92$) than the earthquakes ($M = -0.39$, $SD = 2.76$), which supported Hypothesis 1. The results also revealed a significant type of negative event × geographical proximity interaction, $F\ (1, 34) = 21.72$, $p < .001$, $\eta_p^2 = .390$. The threat appraisal of the earthquake in Asia ($M = 0.11$, $SD = 2.83$) did not differ from the threat appraisal of the terrorist attack in Asia ($M = -0.40$, $SD = 3.14$; $M_{diff} = 0.51$, $SE = 0.52$, $p = .332$). In contrast, the terrorist attack in Europe elicited more threat appraisal ($M = 1.40$, $SD = 2.66$) than the earthquake in Europe ($M = -0.89$, $SD = 2.69$; $M_{diff} = 2.29$, $SE = 0.43$, $p < .001$; Fig 2 on the far left), which supported Hypothesis 4a. The main effect of geographical proximity was not significant, $F\ (1, 34) = 1.53$, $p = .225$.

Fig 2. Means and standard errors for the dependent variables threat, anger, sadness, and anxiety for the Wikipedia articles on earthquakes and terrorist attacks that happened in Asia or Europe of Study 2.

Anger

The results revealed a significant main effect of type of negative event, F (1, 34) = 117.46, $p < .001$, $\eta_p^2 = .776$. The terrorist attacks elicited more anger (M = 5.33, SD = 1.68) than the earthquakes (M = 2.62, SD = 1.68; Fig 2 second from the left), which supported Hypothesis 2. There was also a marginal main effect of geographical proximity, F (1, 34) = 4.04, p = .052, η_p^2 = .106. The negative events in Europe elicited more anger (M = 4.14, SD = 1.76) than the negative events in Asia (M = 3.81, SD = 1.60). The interaction was not significant, F (1, 34) < 1, *ns*. Thus, Hypothesis 4b was not supported. Instead, Hypothesis 2 was supported for terrorist attacks in both Asia and Europe.

Sadness

The results revealed a significant main effect of type of negative event, F (1, 34) = 5.29, p = .028, η_p^2 = .135. The terrorist attacks elicited more sadness (M = 5.37, SD = 2.19) than the earthquakes (M = 4.92, SD = 1.93), which was opposite to Hypothesis 3. There was also a significant type of negative event × geographical proximity interaction, F (1, 34) = 25.45, p < .001, η_p^2 = .428. The earthquake in Asia (M = 5.34, SD = 2.04) did not differ from the terrorist attack in Asia (M = 5.11, SD = 2.33) in the elicitation of sadness (M_{diff} = 0.23, SE = 0.26, p = .383). In contrast, the terrorist attack in Europe elicited more sadness (M = 5.62, SD = 2.05) than the earthquake in Europe (M = 4.51, SD = 1.81; M_{diff} = 1.11, SE = 0.21, p < .001; Fig 2 second from the right), which supported Hypothesis 4c, but was still opposite to Hypothesis 3. The main effect for geographical proximity was not significant, F (1, 34) < 1, *ns*.

Anxiety

The results revealed that there were significant main effects for type of negative event, F (1, 34) = 27.27, p < .001, η_p^2 = .445, and geographical proximity, F (1, 34) = 9.23, p = .005, η_p^2 =

.213, which were qualified by a significant type of negative event × geographical proximity interaction, F (1, 34) = 8.02, p = .008, η_p^2 = .191. The terrorist attack in Asia elicited more anxiety (M = 4.36, SD = 1.91) than the earthquake in Asia (M = 3.67, SD = 1.76; M_{diff} = 0.69, SE = 0.29, p = .023). The terrorist attack in Europe elicited even more anxiety (M = 5.19, SD = 2.20) than the terrorist attack in Asia (M_{diff} = 0.83, SE = 0.17, p < .001) and also more anxiety than the earthquake in Europe (M = 3.70, SD = 1.86; M_{diff} = 1.49, SE = 0.21, p < .001; Fig 2 on the far right).

Discussion

The findings of Study 2 showed that the Wikipedia article on the nearby (i.e., European) terrorist attack elicited more threat appraisal than the Wikipedia article on the nearby earthquake, which was not the case when the negative events happened far away (i.e., in Asia). For the elicitation of anger, however, the geographical proximity of the negative event did not matter. The Wikipedia articles on the nearby and the far away terrorist attacks both elicited more anger than the Wikipedia articles on the nearby and the far away earthquakes. Concerning sadness and anxiety, the Wikipedia article on the nearby terrorist attack elicited more sadness and anxiety than the Wikipedia article on the nearby earthquake. There was no difference in sadness for the Wikipedia articles on the far away earthquake or terrorist attacks, but there was a difference in anxiety.

General discussion

The findings of the current research present evidence that Wikipedia articles on terrorist attacks elicit more threat appraisal and anger than Wikipedia articles on earthquakes. In particular, these emotions were elicited while reading as well as after reading these articles. Moreover, Wikipedia articles on terrorist attacks also elicited more sadness and anxiety while reading and after reading them than Wikipedia articles on earthquakes.

This research complements existing research in three important ways. First of all, previous research on emotional content in Wikipedia articles [2-4] used the automatic linguistic text analysis tool LIWC to investigate emotional content in Wikipedia articles. Although widely used [31-34], this tool may fall short in assessing the broader complexity of emotions. The

present research complements such previous research by having the Wikipedia articles coded by individuals who were capable of specifying their emotions. As previous research, the studies presented here found effects for threat appraisal and anger.

Second, the present research closes an important research gap. Previous research has demonstrated that Wikipedia articles written collectively by masses of individuals as well as individually written Wikipedia articles in the laboratory contained more anger-related content in the case of man-made attacks (e.g., terrorist attacks) and mostly more sadness-related content in the case of disasters (e.g., earthquakes, train accidents). The previous laboratory research also demonstrated that merely thinking about a hypothetical man-made attack elicited more anger and a higher threat appraisal than thinking about a hypothetical man-made or nature-made disaster. The present studies bring these two lines of research together by illustrating that such emotions were also elicited both while reading and after reading Wikipedia articles on real negative events. Importantly, the same effects as in previous research were found. As such, the threat appraisal and anger effects occurred both while externalizing information (i.e., contributing information to Wikipedia articles) and internalizing information (i.e., reading and encoding information from Wikipedia articles).

Finally, the present research introduced a new factor that previous research did not consider. That is, it investigated the geographical proximity of the negative events. In line with other research on the media coverage of certain events [36-37], we found differences between the terrorist attack and the earthquake for threat appraisal, sadness, and anxiety when the events had happened close by. This was not the case when the events happened further away. This means that for threat appraisal, sadness, and anxiety, it mattered where the negative event had happened. The closer-by it happened, that is, in Europe, the more emotional and threat reactions it elicited. In contrast, the distance to Asia seemed to neutralize and attenuate the reactions. For anger, however, it did not matter whether the terrorist attacks were close by or further away. They both elicited more anger than close-by or further away earthquakes. In this sense, the distance of the negative event had no neutralizing or attenuating effect on the anger

effects. Rather, the anger effects for man-made attacks seemed to be independent from geographical proximity.

Implications

In the same way as in previous research, the findings of this research imply that appraisal theories of specific emotions [24-26], theories of threat appraisal [27], and influences of emotions on information processing [16-17] can be applied to Wikipedia. Specific characteristics of certain situations, such as the characteristics of a negative event, elicit specific emotions. That is, reading about the blameworthy actions of terrorists in Wikipedia articles elicits anger and threat appraisal. Thus, those theories are relevant for information production in and knowledge acquisition from Wikipedia articles.

The results of the present research extend previous research in an important way, as they also investigated geographical proximity as a factor influencing emotions in Wikipedia. We revealed that geographical proximity has an impact on threat appraisal, sadness and anxiety, while it has no influence on anger. Thus, regarding theories of specific emotions [24-26], not only the situation of the negative event but also the geographical proximity may have to be taken into account to specify the emotional reaction. Moreover, for Wikipedia researchers and the Wikipedia community, it is relevant to know that negative events happening further away may elicit less emotional reactions than events happening closer by. This finding may have practical implications for Wikipedia authors as well as for researchers studying emotions in Wikipedia.

Strengths, limitations, and future research

The present research used and analyzed existing Wikipedia articles and coded them in two studies which complement each other. While Study 1 used various Wikipedia articles and four coders, Study 2 used 4 articles but had numerous coders. In this sense, the studies were well set up and worked well together. They also introduced a new factor, that is, geographical proximity, which previous research had not taken into consideration so far.

Still, some limitations can be discussed. First, in Study 2, the factor geographical proximity was established by having four Wikipedia articles, two about negative events in Asia

and two about negative events in Europe. Although the effects in this study were clear, other research should aim to replicate these findings with more events. Such research could also specify geographical proximity in a more fine-grained way. For example, it could be differentiated between very close and close negative events in Europe, negative events in Asia which happened just outside of Europe, and further away negative events in Asia. This would yield a more detailed picture of the influence of geographical proximity.

Second, the studies of this research were coding studies which used a classical coding approach or a complete within-groups design in the laboratory. Yet, in future research, the current findings should be replicated in controlled laboratory studies with between-groups factors. Such studies would enable researchers to ensure that the present findings also hold for more conservative statistical tests in between-groups designs.

Finally, the current research found effects for emotional reactions while reading and after reading Wikipedia articles. In order to investigate whether individuals' physiological reactions are the same as the reactions our studies demonstrate, future research could use physiological measures. For example, participants could wear EEG caps that assess their brain activity while reading a Wikipedia article about a terrorist attack. Likewise, physiological measures such as heart rate, blood pressure, skin conductance, and pupil dilatation could be highly relevant additional measures in assessing emotional reactions. These and similar measures would certainly be relevant measures in future research.

Conclusion

In sum, the current research has demonstrated that Wikipedia articles on terrorist attacks elicit more anger and threat appraisal than Wikipedia articles on earthquakes. These effects were established with two coding studies that varied the number of coded Wikipedia articles and the number of coders. This research also illustrated that the emotion effects only occur for negative events in Europe and mostly not for negative events in Asia, except for the anger effect which was the same across Europe and Asia. Thus, the type of negative event and

the geographical proximity of the negative event are relevant factors for explaining threat appraisal and emotions in Wikipedia.

Acknowledgements

We wish to kindly thank Maxi Bürkle, Hannah Engelhardt, Laura Hertner, and Lisa Rau for their help with data coding, study preparation, and data collection.

References

1. Ferron M, Massa P. Psychological processes underlying Wikipedia representations of natural and manmade disasters. Proc WikiSym ’12. New York, NY: ACM Press; 2012. Article No. 2. doi: 10.1145/2462932.2462935
2. Greving H, Cress U, Kimmerle J. Anger in Wikipedia: Perceived intentionality and threat appraisal as mediators of anger about man-made attacks. J Appl Soc Psychol. 2019;49: 99–116. doi: 10.1111/jasp.12568
3. Greving H, Kimmerle J, Oeberst A, Cress U. Emotions in Wikipedia: The role of intended negative events in the expression of sadness and anger in online peer production. Behav Inf Technol. 2019;38: 796–806. doi: 10.1080/ 0144929X.2018.1554702
4. Greving H, Oeberst A, Kimmerle J, Cress U. Emotional content in Wikipedia articles on negative man-made and nature-made events. J Lang Soc Psychol. 2018;37: 267–287. doi: 10.1177/0261927X17717568
5. Laniado D, Kaltenbrunner A, Castillo C, Morell MF. Emotions and dialogue in a peer-production community: The case of Wikipedia. Proc WikiSym ’12. New York, NY: ACM Press; 2012. Article No. 9. doi: 10.1145/2462932.2462944
6. Pennebaker JW, Booth RJ, Boyd RL, Francis ME. Linguistic Inquiry and Word Count: LIWC2015. Austin, TX: Pennebaker Conglomerates (www.LIWC.net); 2015.
7. Cress U, Moskaliuk J, Jeong H. Mass collaboration and education. New York, NY: Springer; 2016. doi: 10.1007/978-3-319-13536-6
8. Matei SA, Dobrescu C. Wikipedia's “neutral point of view”: Settling conflict through ambiguity. Inf Soc. 2010;27: 40–51. doi: 10.1080/ 01972243.2011.534368
9. Oeberst A, Halatchliyski I, Kimmerle J, Cress U. Knowledge construction in Wikipedia: A systemic-constructivist analysis. J Learn Sci. 2014;23: 149–176. doi: 10.1080/10508406.2014.888352
10. Thorsen E. Journalistic objectivity redefined? Wikinews and the neutral point of view. New Media Soc. 2008;10: 935–954. doi: 10.1177/1461444808096252

11. Keegan B, Gergle D, Contractor N. Staying in the Loop: Structure and dynamics of Wikipedia's breaking news collaborations. Proc WikiSym '12. New York, NY: ACM Press; 2012. Article No. 1. doi: 10.1145/2462932.2462934
12. Keegan B, Gergle D, Contractor N. Hot off the wiki: Structures and dynamics of Wikipedia's coverage of breaking news events. Am Behav Sci. 2013;57: 595–622. doi: 10.1177/0002764212469367
13. Barrett LF, Lewis M, Haviland-Jones JM. Handbook of emotions. 4th ed. New York: Guilford Press; 2016.
14. Blaney PH. Affect and memory: A review. Psychol Bull. 1986;99: 229–246. doi: 10.1037/0033-2909.99.2.229
15. Bower GH. Mood and memory. Am Psychol. 1981;36: 129–148. doi: 10.1037/0003-066X.36.2.129
16. Bower GH, Forgas JP. Affect, memory, and social cognition. In: Eich E, Kihlstrom JF, Bower GH, Forgas JP, Niedenthal PM, editors. Cognition and emotion. Oxford, UK: Oxford University Press; 2000. pp. 87–168.
17. Forgas JP, Bower GH. Mood effects on person-perception judgments. J Pers Soc Psychol. 1987;53: 53–60. doi: 10.1037/0022-3514.53.1.53
18. Cress U, Feinkohl I, Jirschitzka J, Kimmerle J. Mass collaboration as co-evolution of cognitive and social systems. In: Cress U, Moskaliuk J, Jeong H, editors. Mass collaboration and education. New York, NY: Springer; 2016. pp. 85–104. doi: 10.1007/978-3-319-13536-6_5
19. Cress U, Kimmerle J. A systemic and cognitive view on collaborative knowledge building with wikis. Int J Comput Support Collab Learn. 2008;3: 105–122. doi: 10.1007/s11412-007-9035-z
20. Cress U, Kimmerle J. The interrelations of individual learning and collective knowledge construction: A cognitive-systemic framework. In: Schwan S, Cress U, editors. The psychology of digital learning: Constructing, exchanging, and acquiring knowledge with digital media. Cham, Switzerland: Springer International Publishing AG; 2017. pp. 123–145. doi: 10.1007/978-3-319-49077-9_7

21. Bientzle M, Cress U, Kimmerle J. How students deal with inconsistencies in health knowledge. Med Educ. 2013;47: 683–690. doi: 10.1111/medu.12198
22. Matschke C, Moskaliuk J, Kimmerle J. The impact of group membership on collaborative learning with wikis. Cyberpsychol Behav Soc Netw. 2013;16: 127–131. doi: 10.1089/cyber.2012.0254
23. Oeberst A, Cress U, Back M, Nestler S. Individual versus collaborative information processing: The case of biases in Wikipedia. In: Cress U, Moskaliuk J, Jeong H, editors. Mass collaboration and education. Cham, Switzerland: Springer International Publishing; 2016. pp. 165–186. doi: 10.1007/978-3-319-13536-6_9
24. Clore GL, Huntsinger JR. How the object of affect guides its impact. Emot Rev. 2009;1: 39–54. doi: 10.1177/1754073908097185
25. Clore GL, Schiller AJ. New light on the affect-cognition connection. In: Barrett LF, Lewis M, Haviland-Jones JM, editors. Handbook of emotions. 4th ed. New York: Guilford Press; 2016. pp. 532–546.
26. Ortony A, Clore GL, Collins A. The cognitive structure of emotions. New York, NY: Cambridge University Press; 1988. doi: 10.1017/CBO9780511571299
27. Blascovich J, Tomaka J. The biopsychosocial model of arousal regulation. Adv Exp Soc Psychol. 1996;28: 1–51. doi: 10.1016/S0065-2601(08)60235-X
28. Henriksen CA, Bolton JM, Sareen J. The psychological impact of terrorist attacks: Examining a dose-response relationship between exposure to 9/11 and axis I mental disorders. Depress Anxiety. 2010;27: 993–1000. doi: 10.1002/da.20742
29. Huddy L, Feldman S. Americans respond politically to 9/11: Understanding the impact of the terrorist attacks and their aftermath. Am Psychol. 2011;66: 455–467. doi: 10.1037/a0024894
30. Neria Y, DiGrande L, Adams BG. Posttraumatic stress disorder following the September 11, 2001, terrorist attacks: A review of the literature among highly exposed populations. Am Psychol. 2011;66: 429–446. doi: 10.1037/a0024791

31. Cohn MA, Mehl MR, Pennebaker JW. Linguistic markers of psychological change surrounding September 11, 2001. Psychol Sci. 2004;15: 687–693. doi: 10.1111/j.0956-7976.2004.00741.x
32. Gosling SD, Mason W. Internet research in psychology. Annu Rev Psychol. 2015;66: 877–902. doi: 10.1146/annurev-psych-010814-015321
33. Iliev R, Dehghani M, Sagi E. Automated text analysis in psychology: Methods, applications, and future developments. Lang Cogn. 2015;7: 265–290. doi: 10.1017/langcog.2014.30
34. Tausczik YR, Pennebaker JW. The psychological meaning of words: LIWC and computerized text analysis methods. J Lang Soc Psychol. 2010;29: 24–54. doi: 10.1177/0261927X09351676
35. Raccanello D, Burro R, Brondino M, Pasini M. Relevance of terrorism for Italian students not directly exposed to it: The affective impact of the 2015 Paris and 2016 Brussels attacks. Stress Health. 2018;34: 338–343. doi: 10.1002/smi.2793
36. Adams WC. Whose lives count? TV coverage of natural disasters. J Commun. 1986;36: 113–122. doi: 10.1111/j.1460-2466.1986.tb01429.x
37. García-Gavilanes R, Tsvetkova M, Yasseri T. Dynamics and biases of online attention: The case of aircraft crashes. R Soc Open Sci. 2016;3: 160460. doi: 10.1098/rsos.160460
38. Roy SD. Paris and Beirut: Data suggest how social media shapes the coverage. Medium (blog post); 2015. Available from: https://goo.gl/M8Xi4J
39. Faul F, Erdfelder E, Lang AG, Buchner A. G*Power 3: A flexible statistical power analysis program for the social, behavioral, and biomedical sciences. Behav Res Methods. 2007;39: 175–191. doi: 10.3758/BF03193146
40. Tomaka J, Blascovich J, Kibler J, Ernst JM. Cognitive and physiological antecedents of threat and challenge appraisal. J Pers Soc Psychol. 1997;73: 63–72. doi: 10.1037/0022-3514.73.1.63
41. Sassenberg K, Hansen N. The impact of regulatory focus on affective responses to social discrimination. Eur J Soc Psychol. 2007;37: 421–444. doi: 10.1002/ejsp.358

Appendix

This appendix contains the titles of the 30 Wikipedia articles on earthquakes and the 30 Wikipedia articles on terrorist attacks that we used in Study 1.

30 Wikipedia articles on earthquakes

2010 Baja California earthquake; 2010 Chile earthquake; 2011 Christchurch earthquake; 2010 Canterbury earthquake; 2009 Fiordland earthquake; 2016 Ecuador earthquake; 2010 Haiti earthquake; October 2015 Hindu Kush earthquake; 2015 Illapel earthquake; 2012 Northern Italy earthquakes; 2016 Taiwan earthquake; 2016 Kumamoto earthquakes; 2009 L'Aquila earthquake; 2011 Lorca earthquake; 2011 Myanmar earthquake; April 2015 Nepal earthquake; 2011 Dalbandin earthquake; 2012 Pernik earthquake; 2010 Pichilemu earthquake; 2009 Samoa earthquake and tsunami; 2010 Serbia earthquake; 2013 Lushan earthquake; 2011 Sikkim earthquake; 2009 Sumatra earthquakes; 2012 Indian Ocean earthquakes; 2012 East Azerbaijan earthquakes; 2011 Tōhoku earthquake and tsunami; 2011 Van earthquake; 2011 Virginia earthquake; 2010 Yushu earthquake.

30 Wikipedia articles on terrorist attacks

2015 Ankara bombings; March 2016 Ankara bombing; February 2016 Ankara bombing; 2015 Beirut bombings; Jewish Museum of Belgium shooting; Boston Marathon bombing; 2016 Brussels bombings; Bacha Khan University attack; Garissa University College attack; 2016 Grand-Bassam shootings; June 2016 Istanbul bombing; January 2016 Istanbul bombing; March 2016 Istanbul bombing; 2016 Jakarta attacks; April 2016 Kabul attack; Metrojet Flight 9268; 2015 Kuwait mosque bombing; 2016 Lahore suicide bombing; 2011 Norway attacks; Charlie Hebdo shooting; November 2015 Paris attacks; 2014 Peshawar school massacre; 2016 Pathankot attack; 2015 Sousse attacks; 2015 Sarajevo shooting; 2015 Suruç bombing; 2015 Thalys train attack; Bardo National Museum attack; Westgate shopping mall attack; Zvornik police station shooting.

Figure 1 Click here to download Figure Fig1.tif

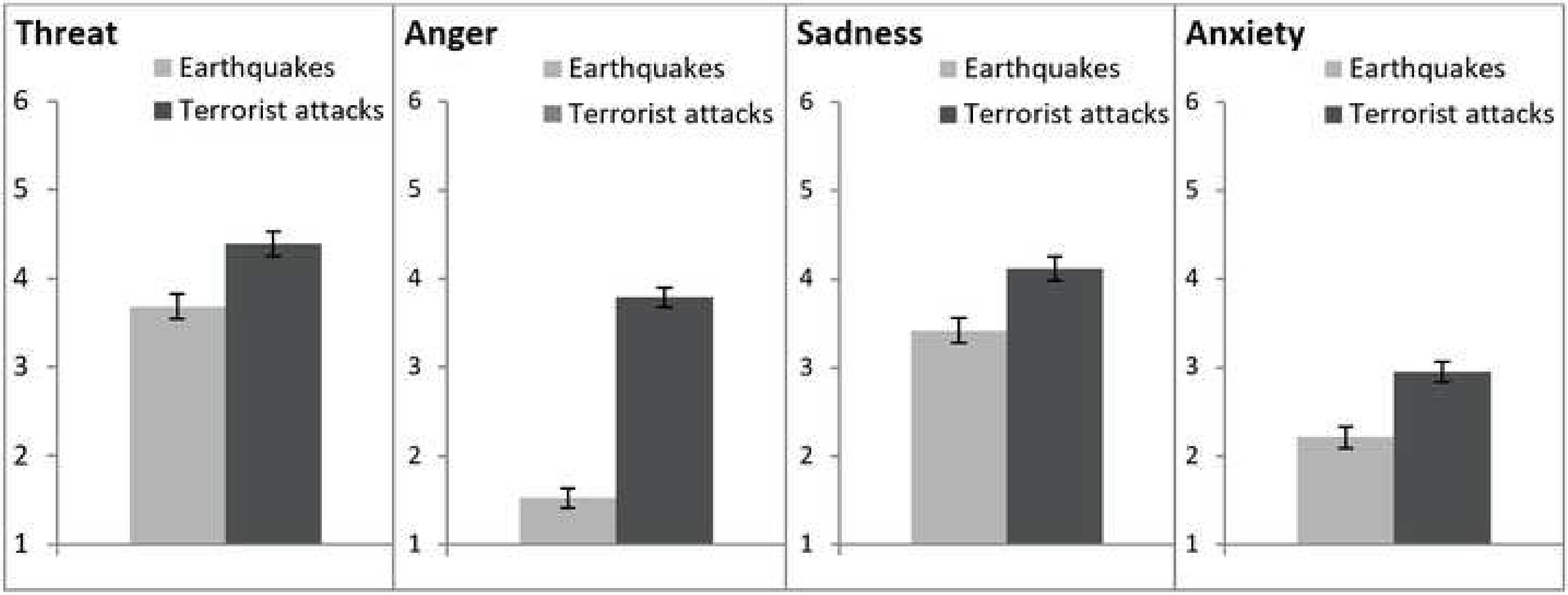

Figure 2

Click here to download Figure Fig2.tif

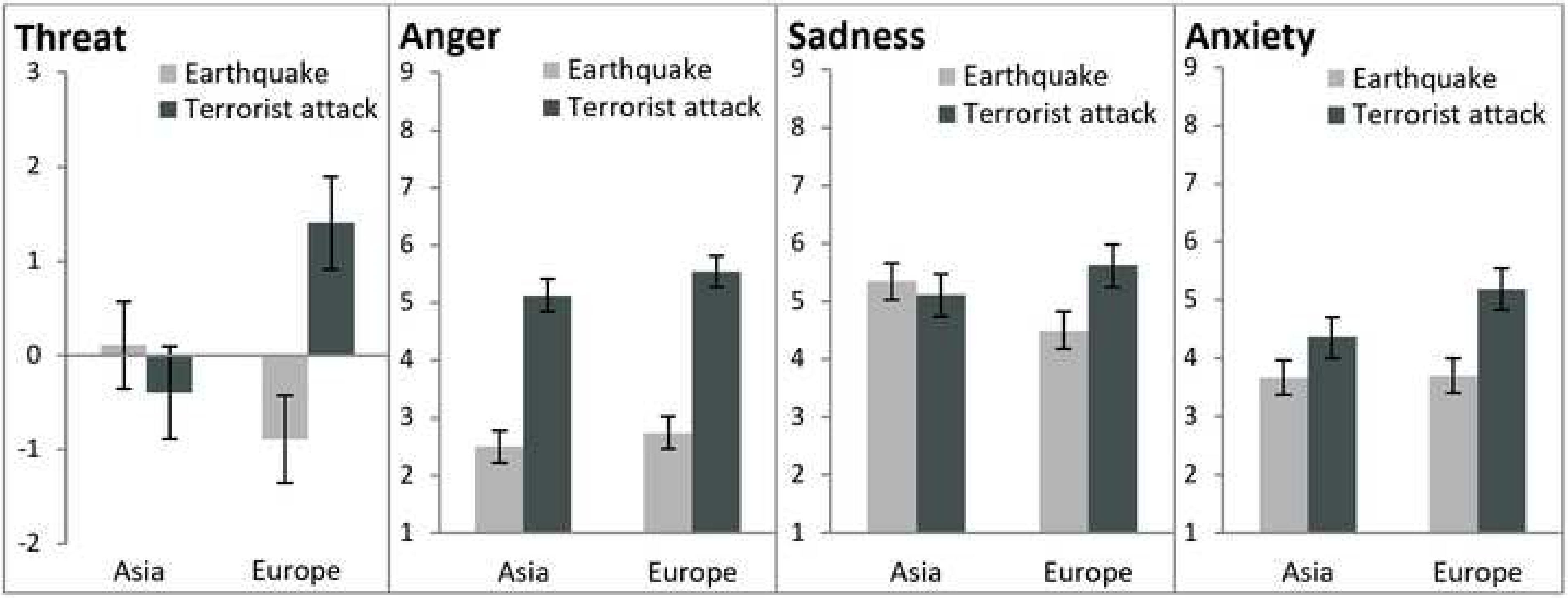

Supplement: Attachment — Submitted filename: PONE-D-19-26386_reviewer copy.highlighted.pdf [file pone.0233770.s005.pdf]
