# Supplementary material for: The impact of event type and geographical proximity on threat appraisal and emotional reactions to Wikipedia articles
Source: PLoS One. 2020 Jun 1;15(6):e0233770. doi: 10.1371/journal.pone.0233770 (PMC7263619; doi:10.1371/journal.pone.0233770)
Supplement: Attachment — Submitted filename: response to reviewers.docx [file pone.0233770.s004.docx]

**Dear Dr. Balas Timar,**

**Thank you very much for investing your time and effort to review our manuscript. Your valuable feedback helped us improve the paper on its way to publication.**

**We have considered all your suggestions and are convinced that we have made appropriate improvements. In this letter, we address all your comments and explain our revisions in detail. In order to ensure the transparency of our revisions, we have replied to each comment separately (in bold letter type). Below our comments, you find the new or modified paragraphs of the revised manuscript (indented, in quotation marks, and in bold letter type).**

Additional Editor Comments (if provided):

The authors present interesting data shedding new light on factors that are relevant for the elicitation of emotions by Wikipedia articles, namely Wikipedia articles on terrorist attacks elicited more threat, anger, sadness, and anxiety than Wikipedia articles on earthquakes.

The authors present to studies. The findings of Study 1 demonstrated that reading Wikipedia articles on terrorist attacks elicited more feelings of threat, anger, sadness, and anxiety than reading Wikipedia articles on earthquakes. The findings of Study 2 showed that the Wikipedia article on the nearby (i.e., European) terrorist attack elicited more threat appraisal than the Wikipedia article on the nearby earthquake, which was not the case when the negative events happened far away (i.e., in Asia).

The manuscript is well written, has an important threat appraisal message, and should be of great interest to the readers.

**Thank you very much. We appreciate your positive feedback to our manuscript.**

However, the results from Study 1 are somehow restrictive in amplitude, due to several aspects:

• “Wikipedia articles about terrorist attacks elicited more feelings of threat (M = 4.39, SD = 0.70) than Wikipedia articles about earthquakes (M = 3.68, SD = 0.84; Fig 1 on the far left), which supported Hypothesis 1”

• “Wikipedia articles about terrorist attacks elicited more anger (M = 3.79, SD = 0.69) than Wikipedia articles about earthquakes (M = 1.53, SD = 0.55; Fig 1 second from the left), which supported Hypothesis 2.”

• Wikipedia articles about terrorist attacks elicited more sadness (M = 4.12, SD = 0.70) than Wikipedia articles about earthquakes (M = 3.42, SD = 0.87; Fig 1 second from the right), which was opposite to Hypothesis 3.

• Wikipedia articles about terrorist attacks elicited more anxiety (M = 2.95, SD = 201 0.69) than Wikipedia articles about earthquakes (M = 2.21, SD = 0.61; Fig 1 on the far right).

when using a 9-point Likert scale ranging from 1 (not at all) to 9 (very much). The means are closer to the non-threat interval (1) rather that being closer to the threat interval (9).

We ask authors to explain such results and to further investigate the meaning of these low means on aspects considered to be important in the investigation of eliciting emotions.

**This is truly a valid point. We have extended the discussion section of Study 1; we state that the means are at the lower end of the scale and we discuss why this could be the case.**

**“The ratings of the independent coders were at the lower end of the 9-point scale. Probably, merely reading about the negative events did not elicit intensive levels of threat, anger, sadness, and anxiety but rather low to medium levels. This may be the case because most of the events had happened in the past and may not have as much influence as new events. Still, the findings clearly demonstrated the expected differences between the negative events. The level and pattern of results also mainly corresponded to previous research [2-4]. Nonetheless, one can easily imagine that the level of emotions would very likely increase in the face of a new terrorist attack or earthquake (e.g., when hearing about a new negative event in the news or when even witnessing or experiencing such an event). Still, the expected difference between terrorist attacks and earthquakes should hold in this case as well.”**

Similar for Study 2, plus the data presenting threat appraisal from lines 292-301 it is inconsistent with results from Study 1. For example “The terrorist attacks elicited more threat appraisal (M = 0.50, SD = 2.92) than the earthquakes (M = -0.39, SD = 2.76), which supported Hypothesis 1.” Means and standard deviations are not properly presented, a mean is not supposed to be negative under the presented methodology. Please revise this section.

**We have also extended the discussion section of Study 2 to address the low to medium levels of anger, sadness, and anxiety.**

**“Similar to Study 1, the scores of the emotions anger, sadness, and anxiety were around the midpoint or lower end of the 9-point scale. As explained in the discussion of Study 1, these moderate scores may have occurred as participants had merely read about the negative events. Possibly, hearing about or watching news on television about new negative events, or even witnessing or experiencing these events may intensely increase these emotions. Still, the difference between earthquakes and terrorist attacks should also hold in such situations.”**

**In the case of the threat appraisal and its results, we apologize for not being clear enough about the measure. We measured threat appraisal with two items and then calculated a difference score between these items, which resulted in a single score for threat appraisal. Therefore, this score could take positive as well as negative values. In order to make up for this shortcoming, we have extended the description of the threat appraisal measure in the methods section of Study 2 and describe in more detail that and how we calculated the difference score.**

**“*Threat appraisal* was measured with two items, a demands item and a resources item. The demands item asked participants “How demanding is the negative event in the article you just read for you?” and assessed their answers on a 9-point Likert scale ranging from 1 (*not demanding at all*) to 9 (*very demanding*). The resources item asked participants “How well can you deal with the negative event?” and assessed their answers on a 9-point Likert scale ranging from 1 (*not at all*) to 9 (*very well*). These items are typical items often used in research about threat [27, 40]. We then calculated a difference score of the two items by subtracting the scores of the resources item from the scores of the demands item, which resulted in a single score for threat appraisal. Thus, this difference score could take positive as well as negative values with higher values indicating more threat appraisal.”**

Overall, it is an important study, and should be considered for publication in PLOS ONE, once the statistical issue has been resolved.

**Thank you very much again for your overall positive feedback. We hope that we have revised the paper in accordance with what you had in mind. Thank you also very much again for the time and effort you invested in reviewing our manuscript. The revisions have improved the manuscript and we think that it is ready for publication.**

**All the best and kind regards,**

**Hannah Greving**
