# Supplementary material for: The impact of event type and geographical proximity on threat appraisal and emotional reactions to Wikipedia articles
Source: PLoS One. 2020 Jun 1;15(6):e0233770. doi: 10.1371/journal.pone.0233770 (PMC7263619; doi:10.1371/journal.pone.0233770)
Supplement: Attachment — Submitted filename: response to reviewers.docx [file pone.0233770.s006.docx]

**Dear Dr. Angel Blanch,**

**Thank you very much for investing your time and effort to review our manuscript. Your constructive and valuable feedback helped us very much improve the paper on its way to publication.**

**We have considered all your suggestions and are convinced that we have made appropriate improvements. In this letter, we address all your comments and explain our revisions in detail. In order to ensure the transparency of our revisions, we have replied to each comment separately (in bold letter type). Below our comments, you find the new or modified paragraphs of the revised manuscript (indented, in quotation marks, and in bold letter type).**

Reviewer #1: This short paper reports on two related studies of emotion expression/perception in Wikipedia articles on earthquakes and terrorist attacks, in a continuation of the authors’ recent research in this area. I agreed to review it because the abstract caught my interest, particular the geographical findings. All the findings are commonsensical and more-or-less predictable, but it can be gratifying to have one’s perceptions confirmed. At first view, the paper appears to be a straightforward and concisely-presented empirical study. It is focused on the research questions, methods, and results of the two related studies, with minimal conceptual framing, review of previous literature, or interpretation beyond confirming or disconfirming the studies’ hypotheses. Although this may be a genre that is acceptable for this journal, I generally see these lacks as shortcomings. In the case of this paper, the abbreviated literature review is most problematic, because readers are not told enough about the authors’ previous work to be able to judge what is original and new in the present work. The References indicate that the authors have published three recent articles (in 2018 and 2019) on what appear to be very closely-related topics. Therefore, the paper needs to make the unique contributions of the present study crystal clear. The geographical question seems to be new in this work; however, the originality of the other research questions is less clear.

**We apologize for having presented only a minimal literature overview of the previous studies and related theories (see below for the other comment concerning the theories). To make up for this shortcoming, we have extended the introduction section of the manuscript and have presented more details about the previous research and the theories:**

**“Second, emotions are core to human beings and influence a wide array of aspects of human functioning and behavior [14]. In particular, they influence information processing [15-16], such as the production of information [17-18], which is relevant for writing Wikipedia articles. Across a variety of contexts, individuals produced more information that was congruent with their current state, for example, more negative information when they were in a negative state [18].” […]**

**“This research used appraisal theories of specific emotions [25-27] to explain the elicitation of certain emotions by certain negative events and used man-made attacks (e.g., terrorist attacks) and natural disasters (e.g., earthquakes) as negative events. These appraisal theories suggest that the characteristics of a situation are important for which emotions are elicited. Emotions can be elicited by desirable or undesirable outcomes, actions, or objects. As we investigate negative events, we will focus on undesirable outcomes and actions. Undesirable outcomes that cannot be made undone and have unspecific causes elicit sadness [25–27]. In contrast, undesirable outcomes that are caused by harmful and blameworthy actions of others who intended to do harm and can be held responsible elicit anger. The previous research mapped these predictions of appraisal theories onto the natural disasters and man-made attacks. In particular, terrorist attacks were expected to elicit more anger, as they occur due to the blameworthy actions of extremists, while earthquakes were expected to elicit more sadness, as they occur due to natural factors and cannot be undone [25-27] (for more elaboration on these expectations see [5]). Following this theorizing and these expectations, the previous research analyzed existing Wikipedia articles in order to investigate whether those expected emotional reactions were present in the manifest content of the articles. It was found that Wikipedia articles on man-made attacks contained more anger-related content than Wikipedia articles on natural disasters, whereas Wikipedia articles on natural disasters contained more sadness-related content than Wikipedia articles on man-made attacks [4-5].**

**Besides the research on the content of existing Wikipedia articles, there has also been research that investigated whether these effects hold when individuals spontaneously produce a Wikipedia article about these negative events. That research found that participants also produced more anger-related content in Wikipedia articles on man-made attacks than in articles on natural disasters in controlled laboratory experiments [3]. These laboratory experiments further examined what could explain the robust anger effects. Based on theories of threat [28], it was argued that man-made attacks elicit threat appraisal and as a consequence anger. Threat appraisal is defined as feelings of being unable to deal with a demanding situation [29]. Threat theories suggest that threat elicits active and engaged reactions, such as increased behavioral intentions and self-serving reactions [28].** **It has also been shown that terrorist attacks were perceived as threatening and increased support for engagement in war activities and aggressive reactions [30–32]. Based on these theories and findings, the previous research has demonstrated that man-made attacks elicited more threat appraisal than nature-made disasters. Threat appraisal was also a mediator that could explain the effects on anger and anger-related content in Wikipedia articles.”**

**Moreover, we have added more information to the introduction in which we point out the contribution of the previous research and especially the contribution of the current research more explicitly:**

**“This previous research investigated which manifest content existing Wikipedia articles about negative events have and which information is produced when spontaneously writing those Wikipedia articles. Thus, the research focused on the expression of emotions in the articles. What has been neglected so far is how information from the Wikipedia articles is received and which emotional reactions are perceived when reading them. These steps are quite important elements in the communication process of Wikipedia, as Wikipedia articles are produced and written for other Internet users who receive the information and perceive its emotional content.” […]**

**“Although widely used, LIWC can be criticized for being based merely on the counts of words. LIWC is not able to assess any context that these emotional words are presented in, any relationships between emotional words, which may hint to totally different emotions (e.g., in the case of sarcasm or irony), or any political or societal meaning of acts or words (e.g., in the case of terrorist attacks). Thus, the results obtained in previous research for the emotional content in the Wikipedia articles may not completely hold, because LIWC may not entirely capture anger, sadness, and anxiety and may not consider the context or other relevant factors. It is also unclear whether individuals reading the Wikipedia articles recognize those emotions that LIWC found and react emotionally in the same way. In order to close this gap, the current research set out to investigate how the content and especially the emotional content of Wikipedia articles on negative events is perceived.”**

I wrote “at first sight” above, because as I read through the paper, I became increasingly aware that two key points are in fact presented somewhat confusingly. The first could be a terminological issue; when I read “the current research coded the emotional content of existing Wikipedia articles” and that the authors “conducted a classical coding study,” I understood that they used content analysis to code emotional expression in the Wikipedia articles. I was surprised to learn later that what they meant was that they had study participants rate their emotional responses to the articles on Likert scales. In my understanding this doesn’t involve “coding,” but rather “rating.”

**We regret that our wording has created a misunderstanding. To address this comment, we have removed the terms “code/coder/coding” from the entire manuscript and instead use “rate/rater/rating”, which are indeed better descriptions of what the raters/participants have done in the studies.**

There is also a deeper underlying confusion that runs through the paper, between production, manifest content, and reception. The authors actually studied the participants’ emotional responses (reception), but they describe it as coding “the emotional content of existing Wikipedia articles” (manifest content). Moreover, later in the paper, they write that “threat appraisal and anger effects occurred … while externalizing information (i.e., contributing information to Wikipedia articles).” This would be fine if Wikipedia contributors had been studied, but they were not; the authors seem to assume that the emotions manifest in the articles are identical to the emotions of the producers of the articles. (The present paper doesn’t analyze the manifest content of the articles, but it seems as though one of their other papers may have done so. Either that, or the authors are assuming that the emotions expressed in the articles are the same as the raters’ emotional responses to the articles, in which case the only emotions for which there is empirical evidence are those of the raters.) In any case, the distinction between production, manifest content, and reception needs to be made clear. Both studies in this paper are concerned primarily with reception.

**These are indeed valid points. First of all, we have given more details of our previous research in the introduction section (see above). This previous research had analyzed the manifest content of Wikipedia articles which is why we had drawn that conclusion. Second, we have added a paragraph to the introduction in which we disentangle and summarize where we have investigated manifest content and the production of information. We also clearly state that the current research deals with the *reception* of information and *perception* of emotional content (see above).**

A third criticism is that the authors describe Study 1 and Study 2 as neatly complementary, “focusing either on many articles with a few coders or a few articles with many coders,” without discussing or even acknowledging in the discussion that the two studies were conducted in different languages, with different participants and different Wikipedia articles. These differences may not ultimately matter much to the studies’ findings, but their possible effects should be considered.

**These are indeed relevant differences. We added a paragraph to the general discussion in which we discuss them:**

**“Still, some limitations can be discussed. First, the articles in Study 1 were in English to ensure comparability with previous research [4–5], while the articles of Study 2 were in German in order to keep the material of the entire laboratory study in German. Such language differences may have caused different results. The raters of the two studies were also different. While they were research assistants collaborating with the authors in Study 1, they were usual laboratory participants in Study 2. Yet, all raters of Study 1 and most raters of Study 2 were university students and therefore comparable. Moreover, the Wikipedia articles were different across the studies. There were more articles in Study 1 than in Study 2. Still, from the four articles of Study 2, one earthquake and one terrorist attack were also used in Study 1. In that sense, there was some overlap in terms of Wikipedia articles between the studies. Besides these differences, the two studies worked well together, but future research should nonetheless strive to minimize possible differences.”**

The paper is written in good, clear English overall. I have highlighted some awkward, nonnative wording in the attached copy of the manuscript.

**Thank you. We have changed these wordings accordingly.**

More detailed comments follow:

L 38 Is www.alexa.com a credible scholarly source? It’s not in the references.

**This website is a web tracking company and, thus, provides reliable statistics about Internet use. To give a more scientific source, we added a reference from the pew research center.**

Ll 56-58 and other statements led me to believe that the content of the articles was coded (using content analysis). But later (e.g., ll 158-160) it seems that it is coders’ emotional reactions that are analyzed, which is not the same thing. Clarify.

**We apologize for the misunderstanding. We have replaced the terms “code/coders/coding” in the entire manuscript with “rate/raters/rating” (see also above).**

Ll 113-114 “LIWC can be criticized for being based merely on the counts of words, without being able to assess broader measures of the complex categories it assesses.” (Vague. What broader measures? For example, one of LIWC’s main weaknesses is its insensitivity to the context in which the words appear, which can affect their meaning.)

**This is absolutely true. We have modified the paragraph about the limitations of LIWC and have added statements about the context problem and two more problematic aspects:**

**“Although widely used, LIWC can be criticized for being based merely on the counts of words. LIWC is not able to assess any context that these emotional words are presented in, any relationships between emotional words, which may hint to totally different emotions (e.g., in the case of sarcasm or irony), or any political or societal meaning of acts or words (e.g., in the case of terrorist attacks). Thus, the results obtained in previous research for the emotional content in the Wikipedia articles may not completely hold, because LIWC may not entirely capture anger, sadness, and anxiety and may not consider the context or other relevant factors. It is also unclear whether individuals reading the Wikipedia articles recognize those emotions that LIWC found and react emotionally in the same way. In order to close this gap, the current research set out to investigate how the content and especially the emotional content of Wikipedia articles on negative events is perceived."**

Ll 127-129 Are you hypothesizing this, or stating it as a fact? If the latter, does that make your research results a foregone conclusion?

**We did not intend to state this statement as a fact. To clarify, we have adjusted these sentences accordingly.**

Ll 133-138 Were the four articles in Study 2 included in the 60 articles Study 1?

**Two articles (April 2015 Nepal earthquake, January 2016 Bacha Khan University attack) were included in the 60 articles in Study 1. In the revised manuscript, we provide this information in the limitations section.**

Ll 144-149 Don’t you already know the answers for some of these Hypotheses from your previous research? What is new about these here?

**This is a valid point. Some of the hypotheses are not completely new. In the revised manuscript we state that directly prior to the hypotheses. What is new is the method with which we have tested the hypotheses. Therefore, before conducting the studies, it was not clear whether they would hold. And in case of sadness (Hypothesis 3), the hypothesis did not hold indeed.**

Ll 158-160 “The elicitation of emotions while reading the articles was measured by having four independent coders rate their subjective emotional reactions during reading.” (Mention this earlier. I misunderstood that the four coders were content analyzing the text of the articles.)

**We regret the misunderstanding. We have added this information in the abstract, the second paragraph of the introduction, and in “the current research” section.**

Ll 170-173 From among all entries that met these criteria for inclusion, how did you choose the 30 (60) for this study? Presumably there were not exactly 30 of each type that met your criteria.

**This is true. We first applied the selection criteria to the lists of earthquakes and terrorist attacks given in Wikipedia. Then, we applied the power calculations. And after that, we chose to use the 30 most recent events from the list in order to ensure timeliness and relevance of the events as well as being safe regarding the power of the study. We have also added this information to the text.**

Ll 190-201 Consistent with your previous research findings? Indicate which of these findings are previously unreported.

**We have added information about which result has been found in some but not other research.**

Ll 226-227 “Study 2 used four existing Wikipedia articles about an earthquake in Asia, an earthquake in Europe, a terrorist attack in Asia, and a terrorist attack in Europe.” (How were these articles selected?)

**We regret that we have not been clearer about how the articles were selected. We have provided more information about the additional selection criteria that we used:**

**“The criteria for the selection of the articles were the same as in Study 1, but we applied the following additional criteria: The articles were available in German, the lengths of the main texts were comparable across geographical location, and the event had happened in the last few years. These additional criteria yielded a sample of roughly 10 articles. From these articles, we then chose those four articles whose length of the German texts was sufficiently long to ensure that participants could engage with the negative events long enough.”**

Ll 236-239 “This analysis showed that we would need a total sample size of 10 participants. We followed these calculations, but simultaneously had to adjust to the conditions in the laboratory and the availability of participants who signed up for the study. Therefore, 35 participants …” (How does it follow that you got 35 if you only needed 10? Explain better.)

**We have explained in more detail for what reasons we had 35 participants.**

**“This analysis showed that we would need a total sample size of 10 participants. We followed these calculations, but simultaneously had to adjust to the conditions in the laboratory and the availability of participants. As participants had signed up for a whole lab session (see below), we could not simply stop the study after the 10 necessary participants. Therefore, more participants than needed participated in this study, that is, 35 participants (26 female, 9 male; *M*_age_ = 26.91, *SD* = 9.94) who had German as mother tongue took part in the study.”**

Ll 241-242 Was the first study in the lab session related to this study?

**The first and the second study were unrelated. The first study had no effects on the dependent measures of the second study.**

Ll 250-252 “The criteria for the selection of the articles …” (Why did you switch from English-language articles in Study 1 to German articles in Study 2? It introduces another possible dimension of variation when comparing the two studies.)

**We provide more elaboration on this point:**

**“We provided German articles as we wanted to keep the language of the laboratory study entirely in German. Moreover, the German articles may have been more easily comprehensible for participants.”**

**Moreover, we chose German as language as participants were to rate their emotional reactions after reading the text. By giving the texts in German, it was more likely that they remembered what they had read about and could more easily provide their emotional reactions.**

Ll 254-256 How were these specific four articles selected from among all candidates that met your inclusion criteria?

**We have provided more information about the additional selection criteria (see above).**

Ll 350-352 “these emotions were elicited while reading as well as after reading these articles. …” (But not the same articles, in the same language, or by the same readers. Discuss.)

**We have discussed these differences in the general discussion (see above).**

L 356 “The broader complexity of emotions” is vague. What do you mean by this, and how do your methods address emotional complexity? It seems that the same emotions are addressed as in previous research, and they are rated as conceptual wholes, without nuance as regards context, individual differences, etc. Expressing vs. perceiving emotions are complementary aspects of communication, to be sure, but do you mean to imply that emotions that are are both expressed and perceived are more “complex” than emotions are are expressed?

**We agree that this term was indeed confusing. We have removed it from the paper. In the introduction, we now describe more precisely what LIWC may not be able to measure and pick that up again in the beginning of the general discussion. In the introduction, we have added a paragraph in which we refer to the distinction between expressing and perceiving emotions and their relevance for communication processes in Wikipedia.**

Ll 370-371 “threat appraisal and anger effects occurred both while externalizing information (i.e., contributing information to Wikipedia articles) and internalizing information (i.e., reading and …” (Careful of your assumptions. What Wikipedia authors write is not necessarily the same as what they feel. You cannot simply equate the two.)

**This is true and we did not mean to equate them. What we meant was the combined effects of the current research and the previous research. We have added this information and explain it more explicitly.**

Ll 384-385 “the anger effects for man-made attacks seemed to be independent from geographical proximity.” (Why might this be so? Is terrorism more “global” than earthquakes, perhaps, in an era where terrorists increasingly travel to wreak havok in other countries?)

**We are grateful for this valuable idea. We have added it to the text:**

**“Possibly, the fact that extremists increasingly aim to do harm across the whole world makes terrorist attacks a more global phenomenon than earthquakes that are tied to specific regions. These differences may have caused the particular anger effects.”**

Ll 388-389 “appraisal theories of specific emotions [24-26], theories of threat appraisal [27], and influences of emotions on information processing [16-17]” (These theories should be laid out more clearly early in the paper, rather than simply referenced.)

**We agree. We describe these theories in more detail in the introduction now (see above).**

Ll 404-405 “The present research used and analyzed existing Wikipedia articles and coded them in two studies …” (Wording like this has caused me confusion throughout the article. You did not code the articles; you had readers rate their emotional responses to the articles, as I now understand. Clarify this throughout. The problem centers on the verb ‘code.’)

**We regret that we have caused this confusion. We refer to it as rating now throughout the entire manuscript (see also above).**

Ll 405-407 Your discussion and conclusions do not acknowledge the differences between the studies (different articles, different language of articles, different readers, different method of recording reactions, etc.). These should be discussed.

**We have provided a discussion of these differences in the general discussion (see also above).**

Ll 417-418 “the studies of this research were coding studies which used a classical coding approach” (I understand this as referring to content analysis, the classic coding and counting approach for analyzing textual and other content. What you did was conduct experiments and have participants rate their emotional responses to text.)

**We did not intend to create confusion about what we did. We have removed the terms “code/coder/coding” from the manuscript and refer to it as “rate/rater/rating” now (see also above).**

Ll 428-429 “These and similar measures would certainly be relevant measures in future research.” (Sentence is rather weak. What additional information might such studies provide? My guess is they will show the same patterns.)

**This is a valid point. We have deleted this sentence and added the following sentence:**

**“If such future research found similar results as we did, it could strengthen the current findings and, therefore, provide further relevant physiological insights about emotional reactions to Wikipedia articles about negative events.”**

**Thank you very much again for your overall positive feedback. We hope that we have revised the paper in accordance with what you had in mind. Thank you also very much again for the time and effort you invested in reviewing our manuscript. The revisions have improved the manuscript and we hope you agree with us that it is ready for publication.**

**All the best and kind regards,**

**Hannah Greving**
