# Supplementary material for: The impact of event type and geographical proximity on threat appraisal and emotional reactions to Wikipedia articles
Source: PLoS One. 2020 Jun 1;15(6):e0233770. doi: 10.1371/journal.pone.0233770 (PMC7263619; doi:10.1371/journal.pone.0233770)
Supplement: Attachment — Submitted filename: response to reviewers.docx [file pone.0233770.s007.docx]

**Dear Dr. Blanch, dear Dr. Herring,**

**Thank you very much for investing your time and effort to re-review our manuscript. Your constructive and valuable feedback again helped us very much improve the paper.**

**We have considered all your suggestions and explain our revisions in detail. In order to ensure the transparency of our revisions, we have replied to each comment separately (in bold letter type). Below our comments, you find the new or modified paragraphs of the revised manuscript (indented, in quotation marks, and in bold letter type).**

Reviewer #1: Most of the points I raised have been cleared up through the authors’ revisions. However, one conceptual confusion persists. Are the raters in Study 1 rating the emotion expressed in the articles (let’s call this A), or rating their own emotional response (call this B)? This confusion is present right from the beginning in the Abstract and the Introduction. Compare, for example, the following two quotes:

Ll 53-55 – “and whether the emotional content in the Wikipedia articles also elicits similar emotional reactions in its readers while and after reading the articles” (B)

Vs.

Ll 58-61 – “Therefore, the current research rated the emotional content of existing Wikipedia articles — with either few raters rating many Wikipedia articles or many raters rating few Wikipedia articles ...” (A)

In other places, (A) and (B) are treated as equivalent in the same text, e.g.,

Ll 212-213 “In order to rate the emotionality of the articles (A), we used four independent raters. Their task was to read each article and rate their own emotional reactions (B) while reading”

(A) and (B) are different. Readers’ emotional reactions are not the same as their perceptions of what emotions are expressed in the text. In fact, after reading the rest of the article, it becomes clearer that only (B) was investigated. To study (A), the authors would have had to have the raters rate the emotion expressed in the text, without regard to their own emotional reactions, e.g., as a content analysis.

Yet it seems the authors also want to claim (A), as a check/confirmation on a previous study that used LIWC to quantify the emotions expressed in the articles themselves. See, e.g.,

Ll 421-423 “The present research makes up for [LIWC’s] shortcoming and complements such previous research by having the Wikipedia articles rated (A) by individuals who were capable of taking those additional factors into account”

(A) would have directly complemented the LIWC study. But that is not what was done in this study. Therefore, all wording that implies that the authors’ did (A) should be changed or omitted.

**We are grateful for the constructive feedback on the conflation between content coding (A) and emotional reactions to content (B). We regret that we have created confusion regarding this point. In the revised manuscript, we have pointed out very clearly that this is an article about emotional reactions to content. We went carefully through the entire manuscript and revised those parts that could cause confusion. We have also revised the statements you quoted above (see also below).**

Other comments:

L 83 – “a variety of contexts, [studies have shown that] individuals produce[d  0] more information that was congruent with their …”

L 87 – “were reflected” --> “are reflected”

L 98 “cannot be made undone” --> “cannot be undone”

**We have changed these sentences accordingly, thank you very much.**

Ll 129-130 “how information from the Wikipedia articles is received and which emotional reactions are perceived when reading them.” ( (A?) Or do you mean, which emotional reactions are produced? That would be (B))

**We meant indeed the emotional reactions that are elicited when reading the articles. We have rephrased this statement accordingly.**

Ll 147-148 “unclear whether individuals reading the Wikipedia articles recognize those emotions that LIWC found (A) and react emotionally (B) in the same way. (These are two separate things.)

**This is true. We have changed the sentence accordingly and only refer to the case that individuals reading Wikipedia articles react emotionally in the same way as indicated by LIWC.**

Ll 153-155 “only English Wikipedia articles were analyzed. This does not allow for taking into account how close or distant the negative event had been for the Internet users who contributed to these articles” (Why not? Because English is used around the world? Explain.)

**We regret that we have not been clear enough concerning this point. We have explained in more detail that anyone who can write in English can contribute to the English articles, meaning that the language of the articles does not indicate where the negative event had happened:**

**“****In this previous research, only English Wikipedia articles were analyzed. This means that the articles could have been written anywhere by anyone who speaks** **English. Consequently, English language articles do not allow for taking into account how close or distant the negative event had been for the Internet users who contributed to these articles.”**

Ll 166-169; 192-194 Focus is squarely on (B) here. Fine; that is what you did.

**This is true. We have done our best to clarify this point throughout the entire manuscript.**

Ll 212-213 “In order to rate the emotionality of the articles (A), we used four independent raters. Their task was to read each article and rate their own emotional reactions (B) while reading” (You did not do (A).)

**We have rephrased the sentence and clearly state that the raters rated their emotional reactions to the articles.**

Ll 306-311 (Mention here that two articles were also in Study 1 (in English)?)

**We have provided this information at the end of this paragraph.**

Ll 421-423 “The present research makes up for [LIWC’s] shortcoming and complements such previous research by having the Wikipedia articles rated (A) by individuals who were capable of taking those additional factors into account” (This study does not directly complement the LIWC study; that would be study (A), having readers rate the emotion expressed in the text (without regard to their own emotions).)

**This is a valid point. We have removed the claim to directly complement the LIWC study.**

Ll 429-432 “The previous laboratory research also demonstrated that merely thinking about a hypothetical man-made attack elicited more anger and a higher threat appraisal than thinking about a hypothetical man-made or nature-made disaster.”

(If I understand what you wrote about this study earlier, you did not measure their emotional response directly, but rather you had participants write Wikipedia articles in which you measured the expressions of emotion, and from which you inferred their emotions indirectly. This is perhaps a subtle distinction, but it illustrates how you conflate emotional reaction and emotional expression. In the same individual, it is relatively unproblematic to equate the two. But in this study it seems you are trying to equate the emotional reactions of your participants with the emotional expression in articles written by others (which you are assuming is the same as their emotional reactions). This is less straightforward.)

**We apology for this misunderstanding and indeed also think that the distinction between emotional reaction and emotional expression is important. The laboratory studies also assessed participants’ emotional reactions and threat appraisal with self-reported measures in addition to the emotional content in the Wikipedia articles written by the participants. To make up for it, we have added this crucial information in the introduction as well as in the discussion:**

**“That research found that participants also produced more anger-related content in Wikipedia articles on man-made attacks than in articles on natural disasters in controlled laboratory experiments [3]. These laboratory experiments also assessed participants’ emotional reactions with self-report measures and further examined what could explain the robust anger effects, both as content in the Wikipedia articles and as self-reported reaction. Based on theories of threat, […]. Based on these theories and findings, the previous research has demonstrated that man-made attacks elicited more threat appraisal than nature-made disasters. Threat appraisal was also a mediator that could explain the effects on anger and anger-related content in Wikipedia articles.”**

**“The previous laboratory research also measured emotional reactions and threat separately with self-report measures and demonstrated that merely thinking about a hypothetical man-made attack elicited more anger and a higher threat appraisal than thinking about a hypothetical man-made or nature-made disaster.”**

Ll 435- 438 “the threat appraisal and anger effects occurred both while externalizing information (i.e., contributing information to Wikipedia articles) [3–5] and internalizing information (i.e., reading and encoding information from Wikipedia articles)” (Yes, this is a more neutral and accurate way to put it.)

**Thank you.**

Ll 486-487 “Still, from the four articles of Study 2, one earthquake and one terrorist attack were also used in Study 1.” (Thanks for clarifying this. It should also be noted in describing your sampling procedure for study 2.)

**We have added this information to the methods section of Study 2, where we described the sampling procedure.**

**Thank you very much again for your constructive feedback. We hope that we have revised the paper in accordance with what you had in mind. Thank you also very much again for your time and effort. The revisions have improved the manuscript and we hope you agree with us that it is ready for publication.**

**All the best and kind regards,**

**Hannah Greving**
